# A High Efficient Biological Language Model for Predicting Protein–Protein Interactions

**DOI:** 10.3390/cells8020122

**Published:** 2019-02-03

**Authors:** Yanbin Wang, Zhu-Hong You, Shan Yang, Xiao Li, Tong-Hai Jiang, Xi Zhou

**Affiliations:** 1 Xinjiang Technical Institutes of Physics and Chemistry, Chinese Academy of Science, Urumqi 830011, China; wangyanbin15@mails.ucas.ac.cn (Y.W.); yangshan16@mails.ucas.ac.cn (S.Y.); xiaoli@ms.xjb.ac.cn (X.L.); jth@ms.xjb.ac.cn (T.-H.J.); zhouxi@ms.xjb.ac.cn (X.Z.); 2 University of Chinese Academy of Sciences, Beijing 100049, China

**Keywords:** protein–protein interactions, bio-language processing, sentencepiece, convolution neural network, unigram language model

## Abstract

Many life activities and key functions in organisms are maintained by different types of protein–protein interactions (PPIs). In order to accelerate the discovery of PPIs for different species, many computational methods have been developed. Unfortunately, even though computational methods are constantly evolving, efficient methods for predicting PPIs from protein sequence information have not been found for many years due to limiting factors including both methodology and technology. Inspired by the similarity of biological sequences and languages, developing a biological language processing technology may provide a brand new theoretical perspective and feasible method for the study of biological sequences. In this paper, a pure biological language processing model is proposed for predicting protein–protein interactions only using a protein sequence. The model was constructed based on a feature representation method for biological sequences called bio-to-vector (Bio2Vec) and a convolution neural network (CNN). The Bio2Vec obtains protein sequence features by using a “bio-word” segmentation system and a word representation model used for learning the distributed representation for each “bio-word”. The Bio2Vec supplies a frame that allows researchers to consider the context information and implicit semantic information of a bio sequence. A remarkable improvement in PPIs prediction performance has been observed by using the proposed model compared with state-of-the-art methods. The presentation of this approach marks the start of “bio language processing technology,” which could cause a technological revolution and could be applied to improve the quality of predictions in other problems.

## 1. Introduction

Protein–protein interactions (PPIs) participate in almost every important life activity, leading to the implementation of various basic functions within the cell. Therefore, a crucial task in the post-genome era is to excavate as many new interaction protein pairs as possible across an entire species. Some high-throughput experimental techniques have been developed to attempt to validate large-scale PPIs, such as two-hybrid systems [1], mass spectrometry [2], protein chip [3], and so on. Since experimental methods are costly in terms of time, money, and labor, the results obtained from these methods is only a small part of the whole PPI network. Furthermore, the results are often accompanied by high false positives and false negatives due to the quality of the experiment being affected by many factors. Therefore, it is of great practical significance to develop reliable computational methods to identify PPIs at low cost and high efficiency.

In recent years, several computational PPI prediction methods have been proposed based on various data sources, such as genomic information, evolutionary knowledge, structural information, and domain information. For example, some methods predict PPIs using phylogenetic profiles [4]. Using the structure of the protein is another popular prediction method. Protein domains are also considered as an information source to predict protein interactions [5] However, these methods cannot be implemented without pre-existing information [6].

Sequence data of proteins is the most available information compared to these sources of information and the potential of proteins primary sequences in inferring PPIs has been well documented. Therefore, sequence-based approaches have attracted wide-ranging concern. Frequently in these methods, the sequence of each protein is represented as a vector using a feature representation method. The vector of protein pairs is then fed into a machine learning algorithm to train a predictor. Thus, protein sequence representation is a core part of the sequence-based approach.

Several popular sequence representation approaches have been reported in PPI prediction methods that have been developed over the past few years. Chou et al. [7] noted that sequence order seems to affect the quality of classification models, so they proposed pseudo-amino acid composition to incorporate the amino acid position effect. Shen et al. [8] have argued that the local environments of amino acids are useful to improve reliability and stability of the prediction model. It is for this reason that they proposed conjoint triad method to consider the effects of the two most adjacent amino acids. Guo et al. [9] reported a method that uses the auto-covariance to account for the interactions between residues that are a long-distance apart in the sequence based on the fact that interactions usually occur in discrete amino acid segments in the sequence. These methods have improved the quality of predicting PPI to varying degrees, however the highest accuracy over any of these methods is still less than 90%. Wang et al. [10,11,12] explored the protein evolutionary features from the angle of the image processing techniques in order to open a new way of researching protein sequences. When performed on a *Yeast* dataset, this method shows good accuracy. However, although this method greatly improves the prediction accuracy of PPI, the use of position-specific scoring matrix tremendously increases the time complexity and computational overhead.

**Figure 1:**
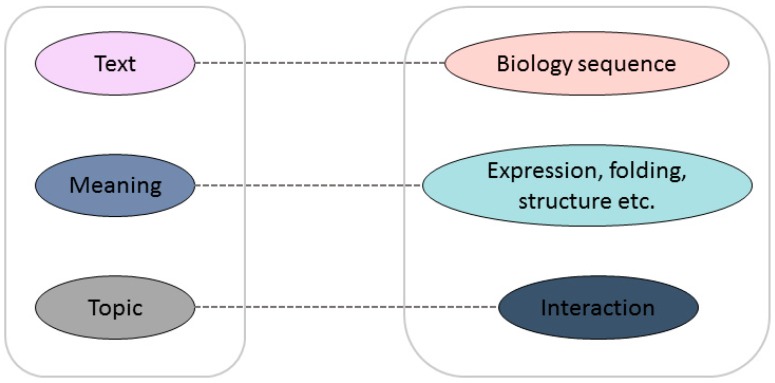
Analogy between natural language and “bio language”.

The major limitation of the prediction capabilities of these methods is that none of them can take account of both local information and the abstract pattern implied in the sequences, even if these methods have their own unique advantages. Inspired by natural language processing, rescanning some fundamental biological problems from a language viewpoint can aid researchers to see the problem from a different perspective in order to find a new solution. Biological sequences, especially protein sequences, can be seen as meaningful genetic languages, which have strong similarities with human language. Researchers have been exploring the link between protein sequences and texts. The mapping relationship between a biological sequence and its structure and function is similar to the word-to-semantic mapping relationship in a language [13]. In languages, words can be arranged into meaningful sentences; in biology, amino acid arrangements determine the structure and function of proteins, which can be viewed as meaningful words to analyze the structure and function of proteins. The similarity is shown in Figure 1. The document maps directly to the semantics and contains relevant information about the topic of the article; similarly, the protein sequence can be regarded as the original text, containing information about structure and function, which can be used to further understand the mutual interaction between proteins. There is no doubt that the development of reliable bio-language technologies to assist in solving biological problems will have a revolutionary impact on current bioinformatics research. The seamless integration of natural language processing technology with biological big data will promote the development of all aspects of the life sciences.

**Figure 2:**
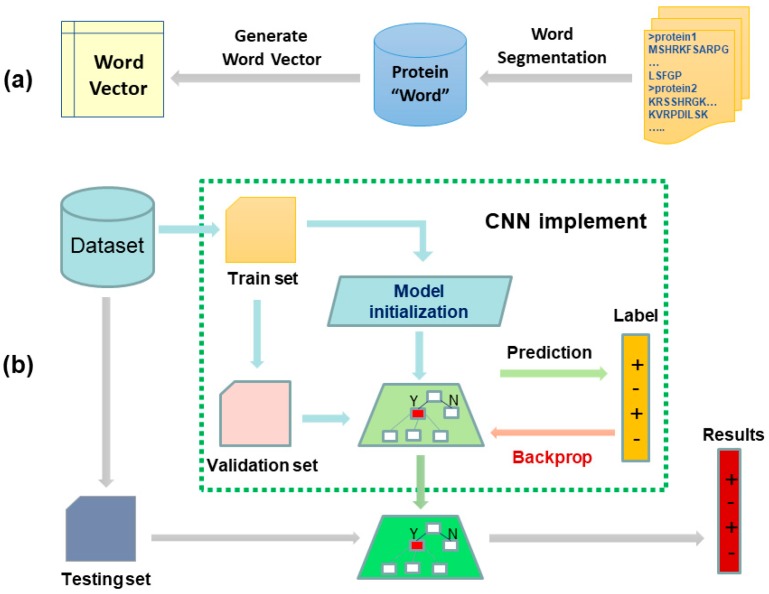
The two-stage workflow of our proposed biology language model for predicting protein–protein interactions (PPIs). Subfigures (**a**) shows the flow of generating fixed-length feature representation for each protein sequence. Given a set of protein sequences, we first segmented them into protein words, and then the protein words were transformed to a vector by Skip-Gram model. Following this, the sequence vector was obtained by accumulating all the protein word vectors of this sequence. Subfigures (**b**) was a convolutional neural network with multiple convolution kernels for predicting PPIs. Given a pair of protein sequences, we represented them using Bio2vec and then concatenated them to form a feature pair. Finally, the trained convolutional neural network was used to predict true or false.

In this paper, a pure bio-language model is proposed for PPIs prediction under the understanding that a protein sequence is similar to text. The model was constructed based on a feature representation method for biological sequences called bio-to-vector (Bio2Vec) and a convolution neural network (CNN). Specifically, we used Bio2Vec to obtain protein sequence features via a “bio-word” segmentation method and Skip-Gram word representation model. Here, the proposed “bio-word” segmentation method was used to segment amino acid sequences into “protein word” sequences, which is based on unigram language model. The Skip-Gram model was used for learning the distributed representation for each “protein word”. Finally, all “protein word” vectors of a protein sequence were integrated to form the feature vector of the protein sequence and fed into a convolution neural network (CNN) for determination of interaction between the two proteins. The flow of our proposed model is represented in Figure 2. The Bio2Vec supplied a frame that allowed us to consider the context information and implicit information in protein sequences, thus, Bio2Vec can cover the local information and semantic information that ran through whole sequence. The results of testing experiments on four gold standard PPIs datasets show that the proposed approach outperforms other state-of-the-art methods. This Bio2Vec was further evaluated by comparison with previous distributed representation methods, the results reveal that the segmentation method has an important impact on prediction quality, supports the biological language hypothesis, and provides a reference for the further study of biological language processing technology.

## 2. Materials and Methods

### 2.1. Data Construction

The first PPI data comes from the *S. cerevisiae* subset of the database of interacting proteins. The reliability of the *S. cerevisiae* data set has been verified by paralogous verification method and expression profile reliability. In order to ensure the validity of the experiment, we strictly followed the work of Guo et al. [8] to collect positive data sets. Twenty-three interacting protein pairs that contain proteins with sequence lengths less than 50 were removed. The 349 protein pairs with more than 40% identity were also excluded, as classifiers may favor these homologous sequence pairs. Finally, the remaining 5594 protein pairs were collected to form a positive data set. We constructed negative datasets by selecting 5594 additional protein pairs with different sub-cellular localizations. The second data set was collected from the Human Protein References Database (HPRD) [14]. We removed those protein pairs that had a greater than 25% sequence identity. Finally, the remaining 3899 experimentally verified PPIs from 2502 different human proteins were collected as the gold standard positive dataset. For the gold standard negative data set, we also followed the assumption that proteins occupying different subcellular localizations do not interact and finally 3899 protein pairs from 661 different human proteins were retained for the construction of the negative data set. The third is the *H. pylori* data set, including 1458 interacting pairs and the same number of non-interacting pairs, that was constructed by following the work of Martin et al. [15]. We established an extended *Human* PPIs data set based on the PICKLE database, which is a meta-database for the human direct protein–protein interactome, integrating publicly available source PPI databases [16]. we arranged 36,630 interacting protein pairs based on the information at the database, and 36,480 non-interacting protein pairs based on the scheme mentioned above.

### 2.2. Bio-Word Segmentation

Processing protein sequences is similar to dealing with the basic building blocks of language. For natural language, these basic units may be letters (such as Hebrew, Greek, English, etc.) or words (such as Chinese, Japanese, etc.). In the absence of language knowledge, dealing with these language problems requires a bottom-up approach: From basic units to vocabulary to semantics and syntax. For biological sequences, the most basic building block is the amino acids or bases. Unlike natural language, the “word” in biological sequence is unknown. To find “biological words”, a data-driven word segmentation algorithm based on unigram language model [17] was utilized to implement automatic word segmentation for the biological sequence. The unigram language model assumes that the occurrence of each word is independent-identically-distribution. The probability of a word segmentation sequence $s=\left(s_{1}s_{2}\ldots s_{N}\right)$ is formulated as the product of the word occurrence probabilities $p\left(s_{i}\right)$:

(1) $$P\left(s\right)=\overset{M}{\underset{i=1}{\prod }}p\left(s_{i}\right)$$

(2) $$\forall i\;s_{i}\in \Phi ,\;\sum_{s\in \mathbb{N}}p\left(s_{i}\right)=1$$

here, Φ is a pre-determined vocabulary. The most probable segmentation $s^{*}$ for the input sentence *R* is then given by

(3) $$s^{*}=\arg maxP\left(s\right),\;s\in X\left(S\right)$$

where X(S) is a set of segmented candidates corresponding to the input sentence *D*. $s^{*}$ is obtained by adopting the Viterbi algorithm [18]. If the vocabulary Φ is known, an Expectation Maximization algorithm [19] can be used to estimate the occurrence probabilities $p\left(s_{i}\right)$. This algorithm maximizes the following marginal likelihood F assuming that $p\left(s_{i}\right)$ are hidden variables.

(4) $$ℱ=\sum_{x=1}^{\left|ℒ\right|}\log\left(P\left(D^{x}\right)\right)=\sum_{x=1}^{\left|ℒ\right|}\log\left(\sum_{s\in X\left(s^{x}\right)}P\left(s\right)\right)$$

where, ℒ is corpus, $D^{x}$ represents the xth sentence in the corpus, $s^{x}$ represents the *x*th candidate. However, the vocabulary for biological sequences is unknown. To find out them, the following iterative algorithm is implemented.

1. We created a reasonable seed vocabulary using the union of all amino acids and the most frequent “amino acid string” in our protein sequence data set. Here, Byte-Pair-Encoding (BPE) algorithm [20] can be used to conduct this step. The BPE first split every protein sequence into individual amino acids. The most frequent adjacent pairs of amino acids were then consecutively merged until reaching a desired seed vocabulary size. Frequent “amino acid string” can be enumerated by the Enhanced Suffix Array algorithm [21], which only takes *O(T)* time and *O(20T)* space.
2. Repeat the following steps until Φ reaches a desired vocabulary size.
  (a) Fixing the set of vocabulary, optimize P(s) by adopting the EM algorithm.
  (b) For each word $s_{i}$, we computed the ${\mathrm{loss}}_{i}$ that measured the change of likelihood ℱ, when the word was removed from the current vocabulary.
  (c) Sort words based on loss and keep top α% (α is 70, in this paper).

We implemented this bio-word segmentation algorithm based on the SentencePiece [22]. The SentencePiece is intended to provide a stable, efficient, and reproducible tool for studying language-agnostic sequences.

### 2.3. Feature Extraction

**Figure 3:**
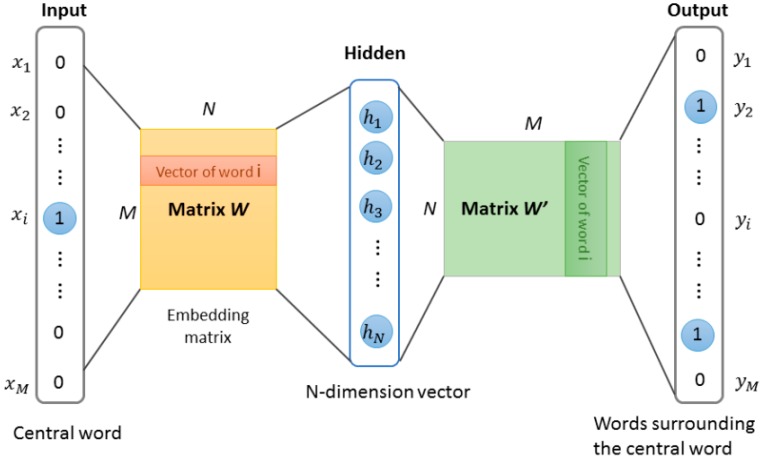
The Skip-gram word representation model. This model is trained by predicting words surrounding the central word. After training, the weights matrix W of the hidden layer was obtained, these weights are actually the “word vectors”.

Our approach for learning high quality fixed-length feature representations from variable-length protein sequences is inspired by the methods for learning the word vectors in natural language processing. The inspiration is that the word vector affects the appearance of the next word in the sentence. Therefore, the learned vector representation can eventually capture semantic knowledge as an indirect result of predicting the next word. We used this idea in this work and implemented protein word representations using the Skip-Gram model [23,24]. The model was essentially a neural network with a projection layer for finding word representations that was useful for the prediction of surrounding words. The structure of Skip-Gram is shown in Figure 3. According to the idea, through three stages, the features of protein sequence can be obtained.

The first stage: Training model to obtain network weights. Given a protein sequence that has been segmented $w_{1},w_{2},\ldots$, $w_{T}$ The goal of the training model is to maximize the following average log probability

(5) $$\frac{1}{T}\sum_{t=1}^{T}\sum_{-c\leq j\leq c,j\neq 0}\log P(w_{t+j}|w_{t})$$

where c indicates the distance from the center word. The definition of P based on the softmax function as follow:

(6) $$\log P\left(w_{O}|w_{I}\right)=\log\frac{\exp\left(v_{w_{O}}^{\prime }{}^{T}v_{w_{I}}\right)}{\sum_{w=1}^{W}\exp\left(v_{w}^{\prime }{}^{T}v_{w_{I}}\right)}$$

where $v_{w}^{\prime }$ and $v_{w}$ are the “output” and “input” *n*-dimensional vector representations of word *w*, respectively. *W* is the size of the protein lexicon (All protein words form the lexicon.). In order to reduce the computational overhead, the Negative Sampling technique is used to approximately maximize the $\log P\left(w_{O}|w_{I}\right)$.

The second stage: Getting the word vector from the weight matrix of the hidden layer.

The third stage: Representing protein sequences. A protein sequence is represented by the sum of all its protein word vectors. Thus, the vector of the protein sequence has the same dimensions as the protein word vector.

(7) $$S_{1:N}=\frac{1}{N}[v_{w_{1}}+v_{w_{2}},\ldots ,\;v_{w_{N}}]$$

here, N means that a protein sequence is split into N words. The feasibility of this approach is derived from the additivity of word meaning and word vector [25]. Compared with the concatenation operation, the method of summation can not only retain the semantic information but also greatly reduce the computational overhead and avoid the error caused by padding.

### 2.4. CNN Construction

What kind of model is suitable for handling such protein features? In this study, we have been understanding and analyzing proteins from the perspective of biological language processing. On this basis, constructing a classifier that performs well in text processing is consistent with the theoretical analysis. Following CNN latest impressive performance in text-classification [26,27,28,29], the PPIs prediction model was constructed based on CNN.

Assume that the protein sequence *S* is represented as a *K*-dimensional feature vector. A convolution operation involves a filter with a window size of h to generate new features. For example, a feature $C_{i}$ is generated from a window of vector elements $S_{i:i+h-1}$ by

(8) $$C_{i}=F\left(w\cdot S_{i:i+h-1}\right)+b$$

where *F* is non-linear function, *w* is filter, *b* is bias. The role of the filter is generating the feature map from protein vector

(9) $$C=[c_{1},c_{1},\ldots ,\;c_{K-h+1}]$$

Then, the max pooling operation is applied over the feature map, and the maximum value *c* = max{*c*} is choose as the feature corresponding to this particular filter. The advantage of this idea is that it retains the most important features of each feature map and greatly reduces the computational complexity of the model.

**Figure 4:**
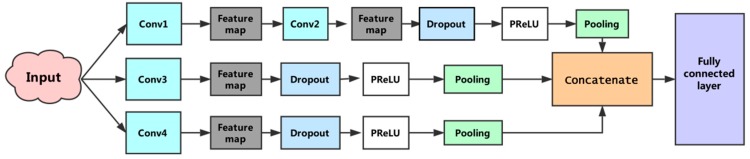
The proposed convolutional neural network architecture. This convolutional neural network consists of three subnetworks. The first sub-network performs two convolutions, the second and third sub-networks perform a convolution, separately, and the fourth convolution operations use different sizes convolution kernels. The penultimate layer is responsible for concatenating features generated by the three subnets. The full connected layer is used to execute the prediction.

In the previous section we described the process of extracting a feature from a filter. In this model, multiple filters with different window sizes were used to obtain multiple features. These features were aggregated at the penultimate layer and passed to the fully connected layer to output a probability result. To prevent overfitting, the dropout [30] operation was applied to the model. Dropout randomly removed units from the neural network and their connections to prevent co-adaptation of hidden units. For the training of the model, we used: Nadam optimizer, a dropout rate of 0.5, early-stopping, and mini-batch size of 64. Finally, the first subnetwork reduced the original feature to 64 dimensions, the second and third sub-networks reduced it to 32-D and 128-D, respectively. Integrating the output of the three subnets, the final feature dimension was 224. Figure 4 shows the architecture of the constructed convolutional neural network. 

For each dataset, nine-tenths from whole dataset were randomly chosen as the training set and the validation set, where the training set accounts for 80% of the extracted data and the verification set for 20%. The training set used for fitting a prediction model and the validation sets used for optimizing the model parameters, the remaining two-tenths were used as test sets for verifying the performance. Several criteria: Accuracy (Accu), precision (Prec), sensitivity (Sens), Matthews’s correlation coefficient (MCC), Receiver operating characteristic (ROC), and Area Under Curve (AUC) were used to comprehensively measure the proposed method. These criteria are sufficient to measure the quality, robustness, and predictability of the model from different perspectives.

(10) $$\mathrm{Accu}=\frac{TN+TP}{FP+TP+FN+TN}$$

(11) $$\mathrm{Sens}=\frac{TP}{TN+TP}$$

(12) $$\mathrm{Prec}=\frac{TP}{TP+FP}$$

(13) $$\mathrm{MCC}=\frac{\left(TN\times TP\right)-\left(FN\times FP\right)}{\sqrt{\left(FN+TP\right)\times \left(FP+TN\right)\times \left(FP+TP\right)\times \left(FN+TN\right)}}$$

where *FP*, *TP*, *FN*, and *TN* represent false positive, true positive, false negative, and true negative, respectively.

## 3. Results

### 3.1. Prediction Performances on Three PPIs Data Sets

**Figure 5:**
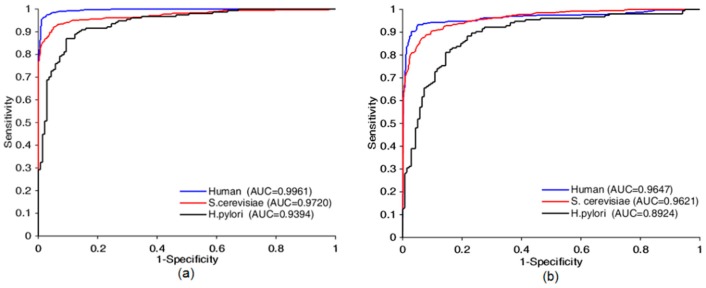
Receiver operating characteristic (ROC) curves comparison of Bio2Vec-based method with 3-mers-based method. ROC curves achieved by Bio2Vec-based method is shown in (**a**), ROC curves achieved by 3-mers-base method is shown in (**b**).

Four prediction models based on the proposed method were built using *Human*, *S.cerevisiae*, *H.pylori* and extended-*Human* data sets, from Table 1 and Figure 5, the AUC was 0.9961, 0.9720, 0.9394, and 0.9995, respectively, the prediction accuracy was 97.31%, 93.30%, 88.01%, and 99.58%, respectively, the precision was 98.48%, 93.55%, 87.90%, and 99.50%, respectively, the sensitivity was 96.28%, 92.70%, 89.61%, and 99.64%, respectively, the MCC was 94.76%, 87.49%, 78.71%, and 99.16% respectively. The false positive on the four datasets were 6, 35, 19, 18, respectively. The false negative on the four datasets were 15, 40, 16, 13, respectively. Table S1 shown some other details about this test experiment. These statistics indicate that our approaches yielded encouraging results. Prediction quality increased with the amount of data used for training. Thus, our model had good scalability and can be further improved by increasing the size of training data sets. The performance suffered when the Skip-Gram word representation model and the designed CNN were applied to small data sets. Our approach still yielded acceptable results, 0.9394 AUC on the small-scale data set (*Matine* data), because the use of the word segmentation method made the semantic information of the sequence fully exposed, which counterbalanced the shortage of training data. To summarize, good results were achieved because we considered the protein sequence as a sentence composed of protein words from the perspective of natural language understanding so that we could jump out from the simple sequence and consider it at a more abstract level. This leads to the knowledge that run through the whole sequence can be taken into account.

### 3.2. Comparison of Different Word Segmentation Schemes

To confirm the effectiveness of the proposed bio word segmentation system, we compared it with *k*-mers-based strategies. The method split a sequence through a sliding window with stride s, where K is the size of window. Therefore, the sequence of length L will be split into [$\frac{L-k}{S}$+1] k-mers. For example, by *3*-mers with stride s=1, “KYMWHKDR” will be split into as “KYM”, “YMW”, “MWH”, “WHK”, “HKD”, and “KDR”. In this test, we followed the previous study to set stride as 1 and split protein sequence into *3*-mers (*3*-mers has been proved to be the best [31]). To make it fair, the remaining parts remained the same. Table 1 gives the comparison results using two different word segmentation approach.

Taken all together, our results obtained by the proposed word segmentation method expose an improvement in prediction accuracy compared to the 3-mers-based methods. Specifically, there was approximately a 5% increase in accuracy in comparison with 3-mer-based method both on *Human* and *H. pylori* data set. The marked difference in the prediction quality reveals the interesting argument, that word segmentation directly affects the quality of a prediction, just like text-processing. This naturally inspired the idea to further enhance the overall level of the prediction by improving the word segmentation techniques for biological sequences. In other words, biological sequences are likely to share some features with language. Moreover, this improvement occurs not just on accuracy level, but is also obvious on other evaluation indicators. The full-scale improvement may be attributed to the fact that the protein words found by our proposed method are more effective in expressing semantics. At the basic hierarchical level of biological language, our protein words cover both static category knowledge of amino acid sequence and dynamic rule knowledge of protein word. On this foundation, the amino acids are no longer viewed in isolation, and the global semantic information carried by the protein sequence is directly considered from the semantic level. Another possible argument for defeating 3-mers is that the *K*-mers produce a lot of repetitive amino acids between words, which leads to redundant information and noise. Additional advantages of Bio2Vec is that it greatly reduces the number of protein words compared with *K*-mers method, which minimizes the computational overhead in the training word vector phase.

### 3.3. Comparison with Previous Studies

To further evaluate the quality of the proposed method in PPIs prediction, we compared it with several state-of-the-art methods. Six methods were separately constructed for comparison in *Human* datasets based on five algorithms, including two feature representation schemes involving Linear Discriminant Analysis (LDA) and Auto-Covariance (AC), three classifiers, involving Random Forest (RF), Support Vector Machine (SVM), Rotation Forest (RoF). On the *S. cerevisiae* dataset, seven methods are used for the comparison. The first two were reported by Guo’s work, that combined SVM classifier with two different feature extraction techniques, ACC and AC. The codes 1 to 4 were built by Yang, performing the PPIs prediction based on four different coding scheme protein pairs and K-Nearest Neighbors. The last one was established using ensemble extreme learning machines. There were also four methods that were used as the basis for comparison with our proposed approach, performed on *H. pylori* dataset. Two of them were based on ensemble approach, and the remaining two used phylogenetic bootstrap algorithm and signature products, respectively. The comparison results are shown in the Table 2, Table 3 and Table 4. The accuracy of the proposed method clearly stood out in comparison with that of other several methods on the three data sets. As Table 2 shows, the accuracy of the proposed method clearly stood out in comparison with several other methods. Table 3 shows that several different methods achieved an average prediction accuracy of less than 90% on *S. cerevisiae* dataset, while our approach obtained an average prediction accuracy of 93.30%. Meanwhile, the sensitivity of 92.70% was also far better than those of the other methods. As shown in Table 4, the 88.01% prediction accuracy achieved by the proposed method was much higher than that of other methods. We only used protein sequences, trying to have an abstract understanding to protein sequences at the linguistic level, which not only brings semantic information, but also avoids the limitations and errors brought by prior knowledge. The Bio2Vec provided a reliable basis for the classifier, and semantic knowledge exposed through word segmentation and word vector directly determined the efficiency and accuracy of the classification recognition. There is also the undeniable fact that the CNN-based classifier provided stable and reliable decisions. Another advantage of the proposed method is that the word vector only needs to be trained once and then can be used to generate protein sequences in any given problem.

## 4. Conclusions

In this study, a biological language model was proposed for PPIs prediction using only protein sequences from a biological language perspective. The model was constructed based on a word representation model named Bio2Vec and a CNN. From the perspective of biological language understanding, a protein sequence was characterized by Bio2Vec that covered the surrounding environment of the center word and semantic information in a protein sequence. Therefore, this method can abstract local features of the protein sequence and consider global semantic information. Our results highlight that the method has good performance and can significantly improve the accuracy of the classifier to distinguish unknown samples. Predictably, Bio2Vec may continue to play a significant role in other prediction problems in proteomics. Moreover, we have shown that protein sequences have language features through comparing the proposed word segmentation schemes and *K*-mers. Our research can be seen as a foundation for advancing biological language processing technology and brings the possibility that increased efforts in discovering protein words may further improve the representational capabilities of Bio2Vec. Generally, our method will expand the research paradigm of computational proteomics and establish interesting connections between biological and natural language processing techniques.

## Figures and Tables

**Table 1:** The comparison of Bio2Vec-based method with 3-mers-based method.

Model	Testing Set	Accu (%)	Sens (%)	Prec (%)	MCC (%)	AUC
Bio2Vec-based	*Human*	97.31	96.28	98.48	94.76	0.9961
	*S. cerevisiae*	93.30	92.70	93.55	87.49	0.9720
	*H. pylori*	88.01	89.61	87.90	78.71	0.9394
	*Extended-Human*	99.58	99.64	99.50	99.16	0.9995
*3*-mers-based	*Human*	92.18	86.85	97.77	85.53	0.9647
	*S. cerevisiae*	90.26	88.14	91.65	82.38	0.9621
	*H. pylori*	83.22	89.61	80.70	82.38	0.8924
	*Extended-Human*	98.47	100	96.98	96.99	0.9998

**Table 2:** Performance comparison of different methods on the *Human* dataset.

Model	Accu (%)	Sens (%)	Prec (%)	MCC (%)
LDA + RF [32]	96.40	94.20	N/A	92.80
LDA + RoF [32]	95.70	97.60	N/A	91.80
LDA + SVM [32]	90.70	89.70	N/A	81.30
AC + RF [32]	95.50	94.00	N/A	91.40
AC + RoF [32]	95.10	93.30	N/A	91.10
AC + SVM [32]	89.30	94.00	N/A	79.20
Proposed Method	97.31	96.28	98.48	94.76

**Table 3:** Performance comparison of different methods on the *S. cerevisiae* dataset.

Model	Accu (%)	Sens (%)	Prec (%)	MCC (%)
ACC [9]	89.33	89.93	88.87	N/A
AC [9]	87.36	87.30	87.82	N/A
Code1 [33]	75.08	75.81	74.75	N/A
Code2 [33]	80.04	76.77	82.17	N/A
Code3 [33]	80.41	78.14	81.66	N/A
Code4 [33]	86.15	81.03	90.24	N/A
PCA-EELM [34]	87.00	86.15	87.59	77.36
Proposed Method	93.30	92.70	93.55	87.49

**Table 4:** Performance comparison of different methods on the *H. pylori* dataset.

Model	Accu (%)	Sens (%)	Prec (%)	MCC (%)
Phylogenetic bootstrap [35]	75.80	69.80	80.20	N/A
Boosting [35]	79.52	80.30	81.69	70.64
Signature products [36]	83.40	79.90	85.70	N/A
HKNN [37]	84.00	86.00	84.00	N/A
Proposed Method	88.01	89.61	80.70	78.71

## Supplementary Materials

The following are available online at http://www.mdpi.com/2073-4409/8/2/122/s1, Table S1: The detail on training set, validation set, test set, false negates and false positives on four PPIs dataset.

## Availability

The code: data, word vector, trained predictor and documentation are available at https://figshare.com/s/b35a2d3bf442a6f15b6e.
