# Supplementary material for: A High Efficient Biological Language Model for Predicting Protein–Protein Interactions
Source: Cells. 2019 Feb 3;8(2):122. doi: 10.3390/cells8020122 (PMC6406841; doi:10.3390/cells8020122)
Supplement: Supplementary file 1 [file cells-08-00122-s001.pdf]

Table S1. The detail on training set, validation set, test set, false negates and false positives on four PPIs dataset.

**Testing Set**	**TraD**	**ValD**	**TestD**	**FN**	**FP**
Human	5615	1402	780	15	6
S. cerevisiae	8055	895	1119	40.	35
H. pylori	2099	525	292	16	19
Extended-Human	52627	13157	7326	13	18
